# Assortative Mating Across the Full Spectrum of Mental Disorders: A Nationwide Finnish Register Study

**DOI:** 10.1016/j.bpsgos.2025.100642

**Published:** 2025-10-28

**Authors:** Kateryna Golovina, Mai Gutvilig, Ripsa Niemi, Christian Hakulinen

**Affiliations:** a Department of Psychology, Faculty of Medicine, University of Helsinki, Helsinki, Finland; b Population Research Institute, Väestöliitto, Family Federation of Finland, Helsinki, Finland; c Finnish Institute for Health and Welfare, Helsinki, Finland

**Keywords:** Assortative mating, Cohabitation, Marriage, Mental disorders, Partner resemblance, Population register data

## Abstract

**Background:**

Previous research has shown assortative mating across various psychiatric disorders; however, their definitions of partnership have often been limited, and the timing of relationship formation has been imprecise. In this study, we aimed to comprehensively examine assortative mating across the full spectrum of mental disorders using population-wide register data from Finland that include information on the formation of both marriages and cohabiting unions.

**Methods:**

We used nationwide data on all cohabitations and marriages between 2000 and 2020 from the Finnish Population Register (*n* = 1,271,242 partnerships). Broad and specific categories of mental disorder diagnoses were retrieved from both primary and secondary health care registers in Finland. We calculated tetrachoric correlations between partners’ mental disorder diagnoses, considering only diagnoses received before the start of cohabitation or marriage.

**Results:**

Assortative mating was observed across the full spectrum of mental disorders, with the strongest within-disorder correlations for schizophrenia, psychotic disorders, organic mental disorders, and intellectual disabilities (*r* > 0.50). Moderate correlations were found for mood and anxiety disorders. Adjusting for birth decade and excluding comorbidities slightly attenuated the associations but did not change the overall patterns.

**Conclusions:**

This study suggests that assortative mating is prevalent in mental disorders. Assortative mating may contribute to the transmission and clustering of mental disorders within families, highlighting the importance of considering partner selection in mental health research and policy making.

## Plain Language Summary

Using nationwide Finnish register data on over 1.27 million couples, this study found that people often partner with others who have similar mental health conditions, a pattern known as assortative mating. The strongest similarities were for severe mental disorders such as schizophrenia and psychosis, while moderate similarities appeared for mood and anxiety disorders. These findings highlight how partner selection may contribute to clustering of mental disorders within families.

Partner resemblance refers to the observed similarity between partners in specific traits or behaviors. This similarity can arise from various mechanisms, including assortative mating, convergence, or other mechanisms over time. In this study, we focus on assortative mating (also known as nonrandom mating), which describes individuals’ tendency to select partners who share similar traits. Studying assortative mating is important because it can influence the distribution of health-related traits and conditions in the population (1). It is crucial to accurately document and account for assortative mating because it can introduce bias in population genetic studies (2,3).

Assortative mating has been documented across various characteristics (4), including mental and somatic health (5). A range of mental disorders has been examined, such as attention-deficit/hyperactivity disorder (ADHD), bipolar disorders, autism spectrum disorder (ASD), depression, and substance use disorders, but previous studies were mainly based on cohort samples with healthy volunteer selection bias (6, 7, 8, 9). To date, only 2 studies have relied on population-wide register-based data from Sweden and Norway and explored a broader range of mental disorders (10,11). The strongest within-disorder correlations were found for ADHD, ASD, schizophrenia, and substance use disorders. The between-disorder correlations were also widespread, but the strengths of the associations varied (10,11). Indirect assortment—where people choose partners based on traits correlated with mental disorders—appears to better explain partner similarity in mental disorders than direct assortment, where partners are selected specifically for having similar mental disorders (11).

However, the definition of partnership varies across studies, and it may not always be possible to disentangle convergence (i.e., whereby a disorder may develop in one partner due to the influence of the other) from assortative mating. For example, a study from Sweden defined partnership as either marriage or biological parenthood without requiring the disorder to be diagnosed prior to partnership formation (10). A Norwegian study anchored partnership to parenthood and deduced the beginning of the relationship based on the time of first childbirth (11). These definitions of partnership rely heavily on couples with children, and it should be noted that people with mental disorders are less likely to become parents (12). Therefore, in our study, we examined assortative mating in the total sample to capture broader social and epidemiological patterns of partner selection. Moreover, there is evidence that spousal convergence is common for certain mental disorders, such as substance use disorders (13). Therefore, it is important to distinguish between convergence and assortative mating.

In the current study, we comprehensively examine assortative mating across the full spectrum of mental disorders using both primary and secondary health care register data from Finland. To overcome the limitations of previous research, we apply a broader definition of partnership, including all unions based on cohabitation and marriage. Cohabitation is a widely accepted form of partnership in the Nordic countries. For example, among cohorts born in the 1970s, the median age at first partnership was around 23, while first marriage occurred after age 30 (14). In these countries, the decline in first marriages has not been accompanied by a decline in first partnerships [except for cohorts born in the 1990s or later, where there is sign of decline in both marital and nonmarital partnerships (15)], indicating that cohabitation has largely replaced marriage as the initial form of union. However, nonmarital unions tend to be less stable than marriages (16,17). Also, the shift toward childbearing outside marriage is well established in the Nordic countries. In Finland, about 50% of first births among women born in the 1970s occurred outside marriage (14). Although marriage is no longer a prerequisite for having a first child, many couples marry subsequently, making it an important context for raising children.

To focus solely on the assortative mating process, we include only those people who were diagnosed with a mental disorder before the start of their cohabitation/marriage. Our first objective was to evaluate the magnitude and significance of partner correlations within and between all mental disorders in a population-based sample. Our second objective was to examine the strength of these associations after controlling for birth decade and comorbidities. Moreover, as previous studies primarily relied on couples with children (10,11), we also compared our results with analyses limited to partners with shared children, enabling us to assess whether the associations differ when these restrictions are applied. Finally, because separation or divorce has been linked to an increased risk of mental disorders (18,19), we conducted a sensitivity analysis restricted to couples in which both partners were in their first recorded relationship.

## Methods and Materials

### Study Population

Data on partnerships, as well as on birth year and any shared children, were retrieved from the full Finnish Population Register of Statistics Finland. The dataset includes the beginning and end dates of cohabitations, marriages, and registered partnerships. Cohabitations were defined according to the definition of Statistics Finland as 2 individuals of different sexes and over the age of 18 years who live in the same residence, are no more than 16 years apart in age, and are not siblings (20). However, if a couple shares children, these criteria do not apply (20). This definition ensures that multiple cohabiting partnerships in institutional settings are not possible within our data. All individuals who got married or started cohabitating at some point between January 1, 2000, and December 31, 2020, were included in the study population. The study population was restricted to different-sex partnerships based on each individual’s registered gender as of June 2024 as the data do not cover all same-sex marriages, registered partnerships, or cohabitations. If a couple cohabited/married more than once, the formation date of the first partnership was used. If a person had multiple partnerships, all partnerships were included.

Partnership data were subsequently linked to data on mental disorders diagnosed prior to the formation of the partnership. Mental disorder diagnoses were retrieved from the Care Register for Health Care and the Register of Primary Healthcare Visits of the Finnish Institute for Health and Welfare. Individual-level register linkages were conducted using personal pseudonymized identity numbers, which are assigned to all Finnish residents. The ethics committee of the Finnish Institute for Health and Welfare approved the study plan (THL/184/6.02.01/2023§933). Data were linked with the permission of Statistics Finland (TK-53-1696-16) and the Finnish Institute for Health and Welfare. Informed consent is not required for register-based studies in Finland.

### Measures

Mental disorder diagnoses (ICD-10 F10 to F99 and equivalent ICD-8, ICD-9, and International Classification of Primary Care, 2nd edition [ICPC-2] codes) included inpatient hospital episodes (1970–2020), secondary outpatient visits (1998–2020), and primary health care visits (2011–2020) in Finland. In our study, broad categories of mental disorders across the whole ICD-10 subchapter F were used: organic mental disorders, including dementia (F00–F09), substance use disorders (F10–F19), psychotic disorders (F20–F29), mood disorders (F30–F39), anxiety disorders (F40–F48), behavioral syndromes associated with physiological disturbances and physical factors (F50–F59), personality disorders (F60–F69), intellectual disabilities (F70–F79), developmental disorders (F80–F89), and childhood-onset disorders (F90–F98). We also used several specific categories of mental disorders: schizophrenia (F20), bipolar disorder (F30–F31), depressive disorder (F32–F34), eating disorders (F50), sleep disorders (F51), pervasive developmental disorders (F84), and hyperkinetic disorders (F90). Finally, we also examined any mental disorder (F00–F99) as its own category. A person was considered diagnosed if they received their diagnosis prior to the start date of the cohabitation/marriage and undiagnosed if they had no record of a diagnosis or the first diagnosis occurred only after the start of cohabitation/marriage.

### Statistical Analysis

Following the approach in Torvik *et al.* (11), we calculated the prevalence of each mental disorder by whether an individual’s partner had also been diagnosed with the same disorder prior to the start of marriage or cohabitation. Next, we calculated tetrachoric correlations both within and across disorders to evaluate the magnitude and significance of the association between partners’ diagnostic status. To account for potential cohort effects, we calculated partial correlations adjusted for both individuals’ birth decades. To evaluate whether the partner correlations differ in partners who share children and those who do not, we stratified the analyses by whether the couple had children together at any point. Consistent with previous studies (10), we treated all observations as independent, even when individuals contributed to more than one couple during the observation period.

We also conducted 3 sensitivity analyses to evaluate the robustness of the findings. First, as relationship formation tends to happen prior to cohabitation/marriage, we repeated the main analysis with only those who had been diagnosed at least 5 years prior to cohabitation/marriage considered as diagnosed. Second, because separation or divorce has been linked to an increased risk of mental disorders (18,19), we focused on the first recorded relationship for both partners in the couple. Partnership data were available from 1987 onward. To ensure that these relationships represented couples’ first unions, we restricted the sample to those who were under age 18 at the beginning of the data period, meaning that both partners were born in 1970 or later. Third, following the study by Nordsletten *et al.* (10) and to reduce confounding from comorbidities, we repeated the main analysis using only the main ICD-10 subchapter F categories. In this analysis, we excluded women diagnosed with the male partners’ disorder of interest and men diagnosed with the female partners’ disorder of interest. For example, when examining correlations between male mood disorders and female anxiety disorders, this analysis included only men without anxiety disorders and women without mood disorders.

For all correlations, we present findings from analyses where the expected contingency table cell frequencies were ≥5 for all cells, and observed cell frequencies were >0. Where applicable, *p* values were corrected for multiple comparisons using the Bonferroni method, with significance set at .05.

Data management was performed using Stata version 17.0 (21). Statistical analyses, figures, and tables were generated in R version 4.2.2 (22) using the packages *lavaan*, *polycor*, and *tidyverse* (23, 24, 25).

## Results

The study population included 964,017 men and 957,207 women, forming 1,271,242 partnerships. Table 1 shows the prevalence of mental disorder diagnoses among men and women. Before the start of cohabitation/marriage, 15.6% of men and 19.7% of women received a mental disorder diagnosis. Anxiety disorders were the most common in the study population, followed by mood disorders and substance use disorders in men and behavioral and emotional syndromes in women. Table S1 shows the data source for the first diagnosis, indicating whether it was recorded in primary care, secondary inpatient, or secondary outpatient registers. To continue, men entered cohabitation or marriage at age 28 on average (IQR = 23–37) and women at age 26 (IQR = 21–34). The median age at diagnosis for each mental disorder category is shown in Table S2. Overall, 39.1% of the partnerships had children. The prevalence of mental disorder diagnoses among partners with children and without is shown in Table S3. For example, depressive and anxiety disorders were more prevalent among parents compared with nonparents, especially among women (11.06% vs. 6.68% for depressive disorders among mothers vs. women without children, respectively; for anxiety disorders, the corresponding percentages were 12.03% vs. 7.47%).

**Table 1:** Prevalence of Mental Disorder Diagnoses Among Men and Women in the Study Population

Diagnosis	Men	Women
Any Mental Disorder, F00–F99	197,782 (15.56%)	249,868 (19.66%)
Organic Mental Disorders, F00–F09	5210 (0.41%)	4509 (0.35%)
Substance Use Disorders, F10–F19	60,248 (4.74%)	41,221 (3.24%)
Psychotic Disorders, F20–F29	12,534 (0.99%)	15,001 (1.18%)
Schizophrenia, F20	3845 (0.3%)	4298 (0.34%)
Mood Disorders, F30–F39	65,977 (5.19%)	121,079 (9.52%)
Bipolar Disorder, F30–F31	8287 (0.65%)	13,473 (1.06%)
Depressive Disorder, F32–F34	63,072 (4.96%)	118,808 (9.35%)
Anxiety Disorders, F40–F48	81,024 (6.37%)	130,240 (10.25%)
Behavioral and Emotional Syndromes, F50–F59	23,382 (1.84%)	47,682 (3.75%)
Eating Disorders, F50	1214 (0.1%)	19,231 (1.51%)
Sleep Disorders, F51	19,739 (1.55%)	28,183 (2.22%)
Personality Disorders, F60–F69	20,691 (1.63%)	23,030 (1.81%)
Intellectual Disabilities, F70–F79	2672 (0.21%)	2704 (0.21%)
Developmental Disorders, F80–F89	18,733 (1.47%)	13,032 (1.03%)
Pervasive Developmental Disorders, F84	1699 (0.13%)	1060 (0.08%)
Childhood-Onset Disorders, F90–F98	30,418 (2.39%)	33,082 (2.6%)
Hyperkinetic Disorder, F90	10,350 (0.81%)	5311 (0.42%)

Values are presented as *n* (%).

**Figure 1:**
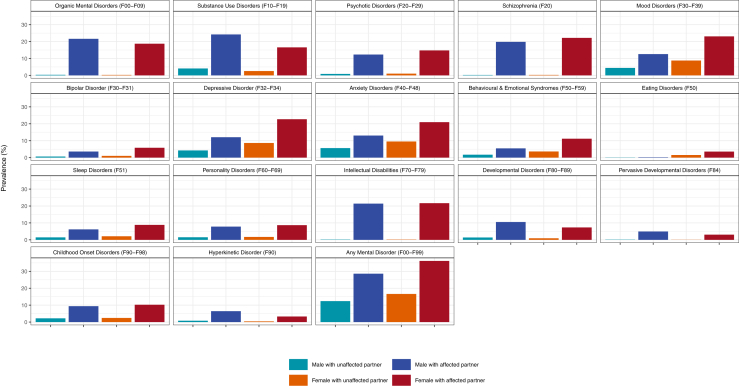
Prevalence of mental disorders among men and women with unaffected and affected partners. Data are shown as the proportion of individuals diagnosed with a specific disorder, stratified by whether their partners have the same disorder.

Figure 1 shows the prevalence of mental disorder diagnoses among men and women with unaffected and affected partners. People with affected partners were more likely to have the same diagnosis themselves, although the strength of this pattern varied by diagnosis. The greatest differences were observed for organic mental disorders (e.g., dementia), substance use disorders, schizophrenia, and intellectual disabilities.

**Figure 2:**
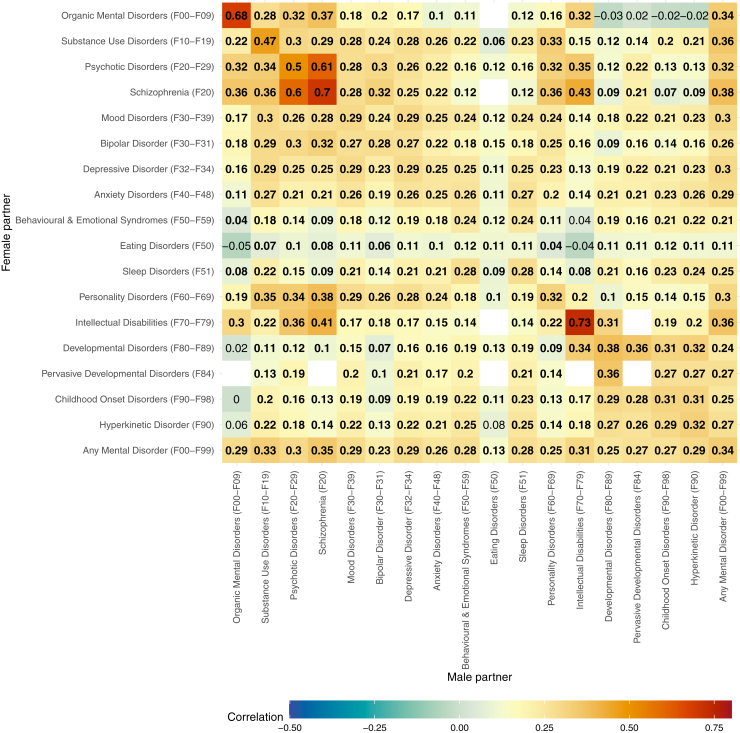
Tetrachoric correlations within and between mental diagnoses of partners. Estimates in bold indicate a statistically significant correlation (Bonferroni-corrected *p* < .05). Empty values indicate that the contingency table had an observed cell frequency of 0 or an expected cell frequency of <5.

Figure 2 shows a heatmap of tetrachoric correlations within and between male and female partners’ mental disorder diagnoses. The strongest within-disorder correlations (>0.50) were found for organic mental disorders, psychotic disorders, schizophrenia, and intellectual disabilities. In contrast, within-disorder correlations for more common mental disorders (e.g., mood disorders, anxiety disorders, and bipolar disorders) ranged from 0.20 to 0.40, with the weakest correlation found for eating disorders (*r* = 0.11).

The strongest between-disorder correlations were observed primarily among severe and early-onset disorders, particularly psychotic and neurodevelopmental conditions (Figure 2). Psychotic disorders and schizophrenia showed the highest cross-partner associations: female partner’s psychotic disorder and male partner’s schizophrenia (*r* = 0.61), and vice versa—male psychotic disorder and female schizophrenia (*r* = 0.60), female schizophrenia and male intellectual disabilities (*r* = 0.43), and male schizophrenia and female intellectual disabilities (*r* = 0.41). Personality disorders in female partners were moderately correlated with a range of male partner diagnoses, including schizophrenia (*r* = 0.38), substance use disorder (*r* = 0.35), male psychotic disorder (*r* = 0.34), mood disorders (*r* = 0.29), bipolar disorder (*r* = 0.28), and anxiety disorder (*r* = 0.24). Similar patterns were observed in the opposite direction—male personality disorders were also linked to female partners’ schizophrenia, substance use, and other disorders. Finally, developmental and childhood-onset disorders were also associated across partners. Female developmental disorders were related to male partners’ pervasive developmental disorders, intellectual disabilities, hyperkinetic disorder, and other childhood-onset conditions. These associations were slightly weaker in the reverse direction but overall indicated similar patterns for male developmental disorders and female partners’ diagnoses.

**Figure 3:**
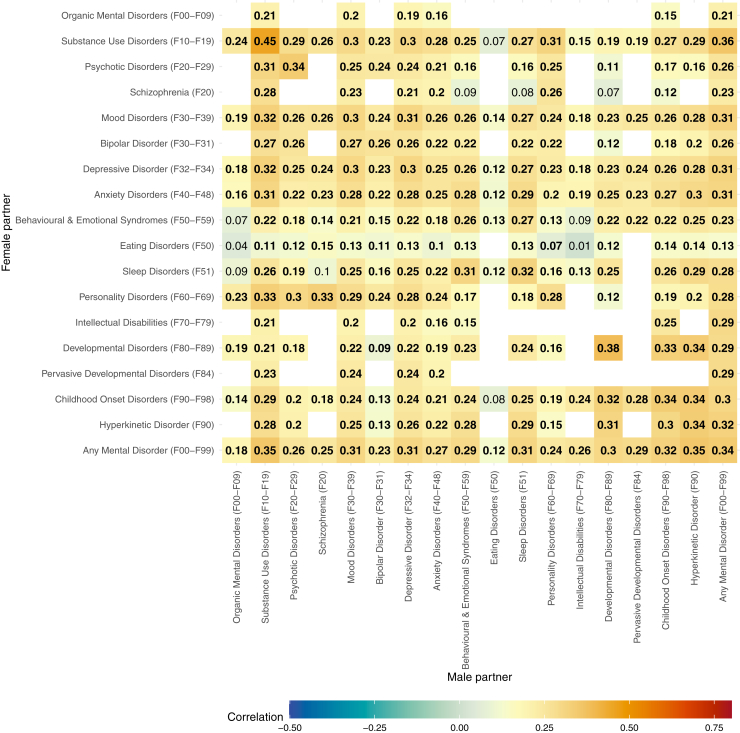
Tetrachoric correlations within and between mental diagnoses of partners with children. Estimates in bold indicate a statistically significant correlation (Bonferroni-corrected *p* < .05). Empty values indicate that the contingency table had an observed cell frequency of 0 or an expected cell frequency of <5.

Figure 3 shows the associations among partners with children. Many associations are not shown due to insufficient data, and the observed associations were slightly attenuated compared with the main model. For example, the associations between female partners’ personality disorders and male partners’ schizophrenia, substance use disorders, and psychotic disorders were reduced compared with the main analysis in the total sample. A similar pattern was observed in the opposite direction, that is, male partners’ personality disorders and the corresponding disorders in female partners. When examining within- and between-disorder associations among partners without children, similar patterns were observed as in the total sample (Figure S1).

The results from sensitivity analyses showed that the associations slightly attenuated after adjusting for birth decade (Figure S2). Similar patterns were observed when considering only diagnoses made more than 5 years before cohabitation (Figure S3). When the analyses were restricted to partners in their first recorded relationship, the results were largely consistent with those in the total sample, although some associations for severe disorders were stronger in the restricted sample (Figure S4). Finally, after excluding comorbidities, the strongest associations were observed between organic mental disorders and intellectual disabilities (*r* = 0.29), organic mental disorders and psychotic disorders (*r* = 0.27), and psychotic disorders and intellectual disabilities (*r* = 0.25). See Figure S5 for details.

## Discussion

This study provides a systematic examination of assortative mating across the full spectrum of mental disorders using nationwide register data from Finland. Our findings are consistent with previous research from Sweden and Norway (10,11), demonstrating strong assortative mating for schizophrenia, intellectual disabilities, psychotic disorders, organic mental disorders, and substance use disorders. Additionally, we observed widespread between-disorder correlations among partners with varying strengths. The adjustments for birth decade, having children, first partnership, comorbidities, and diagnosis made 5 years before cohabitation slightly attenuated the associations but did not change them.

The strong within-disorder correlations for severe mental disorders may suggest that men and women with these diagnoses are particularly likely to form partnerships with others who have the same disorder. This may be explained by shared genetic vulnerabilities, environmental exposures, or social homogamy, where individuals with similar life circumstances and experiences are more likely to meet and form relationships (5). Individuals with severe mental disorders may also reside in psychiatric residential facilities where the selection of potential partners is limited and often includes others with the same diagnoses. Indirect assortative mating may also explain some of the findings, as shown recently (11). Partners may not assort directly on a specific mental disorder but rather on correlated traits, such as personality characteristics or cognitive functioning, which in turn increase the likelihood of shared diagnoses.

For other mental disorders, including mood and anxiety disorders, assortative mating was present but to a lesser extent. These weaker correlations may reflect the higher prevalence of these disorders in the general population and their heterogeneity in symptoms, making assortative mating less pronounced. Nevertheless, significant partner correlations indicate that selection into relationships based on mental health status is not random. It should be noted that organic mental disorders (e.g., dementia) typically have a later average age of onset compared with the timing of marriage and cohabitation and may not be comparable with other mental disorders. However, these disorders were included for completeness and comparability across the full spectrum of mental disorders based on the ICD-10 classification.

Our sensitivity analyses showed that adjusting for birth decade slightly attenuated associations but did not change our findings, indicating that cohort effects do not fully explain partner correlations. When we restricted the analyses to diagnoses made more than 5 years before cohabitation, similar patterns were observed, suggesting that assortative mating occurs prior to partnership formation rather than being driven by shared environmental influences during the early stages of the relationship prior to cohabitation/marriage. Likewise, when we restricted the sample to the first registered partnership for both partners in the couple, the results were consistent with those from the total sample, with some associations for severe disorders being stronger in the restricted sample. Among partners with children, some associations were not presented due to insufficient data, and associations were slightly attenuated. This attenuation could be due to selection effects wherein individuals with severe mental disorders are less likely to have children (12,26,27). However, the general trend of assortative mating remained and is consistent with previous register-based studies from Sweden and Norway that largely relied on couples with children (10,11). Additionally, after excluding comorbidities, the strongest cross-disorder associations were between female partners’ organic mental disorders and male partners’ intellectual disabilities, organic mental disorders, and psychotic disorders, as well as female partners’ psychotic disorders and male partners’ intellectual disabilities. These associations were mirrored in the opposite direction—male partners’ organic mental disorders or psychotic disorders and the corresponding disorders in female partners.

### Strengths and Limitations

The main strength of our study was the use of both primary and secondary health care data from the Finnish nationwide registers, enabling us to examine the associations with high statistical accuracy and minimal health-related selection biases. Combining primary and secondary care data allows to better capture the overall occurrence of different types of mental disorders (28). Register-based mental disorder diagnoses have generally been shown to have good validity (29, 30, 31), but not all diagnoses have been validated; variations have likely occurred across clinical settings (29); and primary care data may be partially incomplete due to technical issues and regional differences (28). Additionally, our broader definition of partnership allowed a more comprehensive examination of assortative mating, as the population register includes complete data on all marriages, cohabitations, and registered partnerships from 2000 to 2020.

However, the current findings should be interpreted in light of several limitations. First, although our data included people treated in both primary and secondary health care settings, the availability of these data varied over time: Inpatient records were available from 1970, outpatient records from 1998, and primary care visits only from 2011. This means that earlier birth cohorts and couples formed before 2011 may have less complete coverage of more common mental disorders, which could lead to underestimation of prevalence in these groups. However, while controlling for birth decade attenuated some associations, the overall patterns remained largely unchanged. Additionally, the data lack information on the onset, remission, and recovery of mental disorders. Therefore, we cannot distinguish whether individuals formed relationships, for example, during the active phases of the disorder or after recovery. Second, we did not have the information on the date of relationship formation, only the date of marriage or cohabitation. To eliminate this limitation, we conducted sensitivity analysis by including only those diagnosed at least 5 years prior to cohabitation or marriage. Third, we did not account for whether marriages or cohabitations occurred earlier, on time, or later relative to Finnish social norms, which may influence the observed associations. Finally, our study was restricted to different-sex partnerships, as same-sex relationships were not systematically recorded in the data. Future studies should examine whether similar assortative mating patterns hold in same-sex couples.

### Conclusions

This study found that assortative mating is prevalent both within and across the full spectrum of mental disorders. The strongest associations were found for severe mental disorders, including schizophrenia, psychotic disorders, organic mental disorders, and intellectual disabilities. Common mental disorders, such as mood and anxiety disorders, showed moderate correlations with each other but also frequently co-occurred with other mental disorders, especially severe ones. While these findings are largely consistent with previous research (10,11), our subgroup analyses suggest that earlier findings may be attenuated due to relying on partners with shared children. Taken together, these findings support the role of assortative mating as one of the mechanisms contributing to the transmission and clustering of mental disorders within families. Future studies should examine the underlying mechanisms driving assortative mating across mental disorders, such as partner selection based on shared psychological traits or experiences or social homogamy through common social and socioeconomic environments. Additionally, examining the long-term consequences of within- and between-trait assortative mating is important for the social and public health implications of these patterns.

## Supplementary Material

Figures S1–S5 and Tables S1–S3

Key Resources Table
