# Supplementary material for: Assortative Mating Across the Full Spectrum of Mental Disorders: A Nationwide Finnish Register Study
Source: Biol Psychiatry Glob Open Sci. 2025 Oct 28;6(1):100642. doi: 10.1016/j.bpsgos.2025.100642 (PMC12719663; doi:10.1016/j.bpsgos.2025.100642)
Supplement: Figures S1–S5 and Tables S1–S3 [file mmc1.pdf]

# SUPPLEMENTARY INFORMATION

# Assortative Mating Across the Full Spectrum of Mental Disorders: A Nationwide Finnish Register Study

Golovina *et al.*

## Contents

| | |
|---|---|
| **Supplementary Table 1.** | Data sources for first diagnosis: primary care, secondary inpatient, and secondary outpatient registers. |
| **Supplementary Table 2.** | Median age at diagnosis. |
| **Supplementary Table 3.** | Prevalence of mental disorder diagnoses among parents and not parents, separately for men and women. |
| **Supplementary Figure 1.** | Tetrachoric correlations within and between mental diagnoses of partners without children. |
| **Supplementary Figure 2.** | Tetrachoric correlations within and between mental diagnoses of partners adjusted for a birth decade. |
| **Supplementary Figure 3.** | Tetrachoric correlations within and between mental diagnoses of partners including only diagnoses 5 years before cohabitation. |
| **Supplementary Figure 4.** | Tetrachoric correlations within and between mental diagnoses of partners in the first recorded relationship. |
| **Supplementary Figure 5.** | Tetrachoric correlations within and between mental diagnoses of partners when comorbidities were excluded. |

**Supplementary Table 1.** Data sources for first diagnosis: primary care, secondary inpatient, and secondary outpatient registers.

| Diagnosis | First Diagnosis Source | Men, n (%) | Women, n (%) |
|---|---|---|---|
| Any Mental Disorder (F00-F99) | | | |
| | primary | 38700 (19.57%) | 69258 (27.72%) |
| | secondary inpatient | 72416 (36.61%) | 50022 (20.02%) |
| | secondary outpatient | 86666 (43.82%) | 130588 (52.26%) |
| Organic Mental Disorders (F00-F09) | | | |
| | primary | 662 (12.71%) | 648 (14.37%) |
| | secondary inpatient | 2407 (46.2%) | 1988 (44.09%) |
| | secondary outpatient | 2141 (41.09%) | 1873 (41.54%) |
| Substance Use Disorders (F10-F19) | | | |
| | primary | 9053 (15.03%) | 6283 (15.24%) |
| | secondary inpatient | 27692 (45.96%) | 15528 (37.67%) |
| | secondary outpatient | 23503 (39.01%) | 19410 (47.09%) |
| Psychotic Disorders (F20-F29) | | | |
| | primary | | 437 (2.91%) |
| | secondary inpatient | 7482 (59.69%) | 8892 (59.28%) |
| | secondary outpatient | 4668 (37.24%) | 5672 (37.81%) |
| | unknown | | 0 (0%) |
| Schizophrenia (F20) | | | |
| | primary | | 98 (2.28%) |
| | secondary inpatient | 2956 (76.88%) | 3276 (76.22%) |
| | secondary outpatient | 801 (20.83%) | 924 (21.5%) |
| | unknown | | 0 (0%) |
| Mood Disorders (F30-F39) | | | |
| | primary | 12925 (19.59%) | 25134 (20.76%) |
| | secondary inpatient | 19172 (29.06%) | 23349 (19.28%) |
| | secondary outpatient | 33880 (51.35%) | 72596 (59.96%) |
| Bipolar Disorder (F30-F31) | | | |
| | primary | 763 (9.21%) | 1325 (9.83%) |
| | secondary inpatient | 3763 (45.41%) | 5037 (37.39%) |
| | secondary outpatient | 3761 (45.38%) | 7111 (52.78%) |
| Depressive Disorder (F32-F34) | | | |
| | primary | 14394 (22.82%) | 28858 (24.29%) |
| | secondary inpatient | 16736 (26.53%) | 20383 (17.16%) |
| | secondary outpatient | 31942 (50.64%) | 69567 (58.55%) |
| Anxiety Disorders (F40-F48) | | | |
| | primary | 20638 (25.47%) | 46265 (35.52%) |

| | | | |
|---|---|---|---|
| | secondary inpatient | 28312 (34.94%) | 17304 (13.29%) |
| | secondary outpatient | 32074 (39.59%) | 66671 (51.19%) |
| Behavioural & Emotional Syndromes (F50-F59) | | | |
| | primary | 15795 (67.55%) | 25087 (52.61%) |
| | secondary inpatient | 3032 (12.97%) | 4977 (10.44%) |
| | secondary outpatient | 4555 (19.48%) | 17618 (36.95%) |
| Eating Disorders (F50) | | | |
| | primary | 88 (7.25%) | 2386 (12.41%) |
| | secondary inpatient | 399 (32.87%) | 3282 (17.07%) |
| | secondary outpatient | 727 (59.88%) | 13563 (70.53%) |
| Sleep Disorders (F51) | | | |
| | primary | 14223 (72.06%) | 23226 (82.41%) |
| | secondary inpatient | 2547 (12.9%) | 1465 (5.2%) |
| | secondary outpatient | 2969 (15.04%) | 3492 (12.39%) |
| Personality Disorders (F60-F69) | | | |
| | primary | 817 (3.95%) | 1683 (7.31%) |
| | secondary inpatient | 12753 (61.64%) | 9838 (42.72%) |
| | secondary outpatient | 7121 (34.42%) | 11509 (49.97%) |
| Intellectual Disabilities (F70-F79) | | | |
| | primary | 162 (6.06%) | 195 (7.21%) |
| | secondary inpatient | 1890 (70.73%) | 1743 (64.46%) |
| | secondary outpatient | 620 (23.2%) | 766 (28.33%) |
| Developmental Disorders (F80-F89) | | | |
| | primary | 1031 (5.5%) | 1572 (12.06%) |
| | secondary inpatient | 4298 (22.94%) | 2183 (16.75%) |
| | secondary outpatient | 13404 (71.55%) | 9277 (71.19%) |
| Pervasive Developmental Disorders (F84) | | | |
| | primary | 76 (4.47%) | 70 (6.6%) |
| | secondary inpatient | 465 (27.37%) | 256 (24.15%) |
| | secondary outpatient | 1158 (68.16%) | 734 (69.25%) |
| Childhood Onset Disorders (F90-F98) | | | |
| | primary | 2610 (8.58%) | 4592 (13.88%) |
| | secondary inpatient | 8791 (28.9%) | 8687 (26.26%) |
| | secondary outpatient | 19017 (62.52%) | 19803 (59.86%) |
| Hyperkinetic Disorder (F90) | | | |
| | primary | 1306 (12.62%) | 1223 (23.03%) |
| | secondary inpatient | 1730 (16.71%) | 574 (10.81%) |
| | secondary outpatient | 7314 (70.67%) | 3514 (66.16%) |

*Note*. To avoid the identification of individual people, data were censored if one of the categories contained less than 3 observations.

**Supplementary Table 2.** Median age at diagnosis.

| Diagnosis | Men, Median (IQR) | Women, Median (IQR) |
|---|---|---|
| Any Mental Disorder (F00-F99) | 21 (16–30) | 20 (16–28) |
| Organic Mental Disorders (F00-F09) | 51 (26–76) | 64 (30–80) |
| Substance Use Disorders (F10-F19) | 26 (20–36) | 22 (18–33) |
| Psychotic Disorders (F20-F29) | 25 (20–33) | 24 (19–31) |
| Schizophrenia (F20) | 26 (22–33) | 26 (21–34) |
| Mood Disorders (F30-F39) | 26 (20–36) | 22 (17–31) |
| Bipolar Disorder (F30-F31) | 30 (23–39) | 25 (20–34) |
| Depressive Disorder (F32-F34) | 25 (20–36) | 22 (17–31) |
| Anxiety Disorders (F40-F48) | 22 (19–30) | 22 (17–29) |
| Behavioral & Emotional Syndromes (F50-F59) | 24 (19–34) | 19 (16–26) |
| Eating Disorders (F50) | 17 (13–21) | 17 (15–21) |
| Sleep Disorders (F51) | 24 (19–34) | 22 (18–31) |
| Personality Disorders (F60-F69) | 23 (19–31) | 24 (20–31) |
| Intellectual Disabilities (F70-F79) | 16 (8–25) | 16.5 (9–27) |
| Developmental Disorders (F80-F89) | 9 (6–12) | 10 (6–15) |
| Pervasive Developmental Disorders (F84) | 12 (8–16) | 14 (10–19) |
| Childhood Onset Disorders (F90-F98) | 12 (8–15) | 14 (11–16) |
| Hyperkinetic Disorder (F90) | 13 (8–19) | 18 (13–25) |

**Supplementary Table 3.** Prevalence of mental disorder diagnoses among parents and not parents, separately for men and women.

| **Diagnosis** | **Parents** | | **Not parents** | |
|---|---|---|---|---|
| | Men, % (n) | Women, % (n) | Men, % (n) | Women, % (n) |
| Any Mental Disorder (F00-F99) | 18.46% (142950) | 22.91% (177465) | 11.04% (54832) | 14.58% (72403) |
| Organic Mental Disorders (F00-F09) | 0.58% (4529) | 0.53% (4087) | 0.14% (681) | 0.08% (422) |
| Substance Use Disorders (F10-F19) | 5.88% (45552) | 3.94% (30524) | 2.96% (14696) | 2.15% (10697) |
| Psychotic Disorders (F20-F29) | 1.27% (9818) | 1.52% (11786) | 0.55% (2716) | 0.65% (3215) |
| Schizophrenia (F20) | 0.43% (3311) | 0.49% (3788) | 0.11% (534) | 0.1% (510) |
| Mood Disorders (F30-F39) | 6.38% (49441) | 11.29% (87456) | 3.33% (16536) | 6.77% (33623) |
| Bipolar Disorder (F30-F31) | 0.84% (6499) | 1.33% (10331) | 0.36% (1788) | 0.63% (3142) |
| Depressive Disorder (F32-F34) | 6.1% (47212) | 11.06% (85633) | 3.19% (15860) | 6.68% (33175) |
| Anxiety Disorders (F40-F48) | 7.35% (56921) | 12.03% (93137) | 4.85% (24103) | 7.47% (37103) |
| Behavioural & Emotional Syndromes (F50-F59) | 2.27% (17557) | 4.38% (33896) | 1.17% (5825) | 2.78% (13786) |
| Eating Disorders (F50) | 0.11% (862) | 1.63% (12632) | 0.07% (352) | 1.33% (6599) |
| Sleep Disorders (F51) | 1.91% (14821) | 2.73% (21116) | 0.99% (4918) | 1.42% (7067) |
| Personality Disorders (F60-F69) | 1.96% (15212) | 2.25% (17400) | 1.1% (5479) | 1.13% (5630) |
| Intellectual Disabilities (F70-F79) | 0.29% (2248) | 0.29% (2255) | 0.09% (424) | 0.09% (449) |
| Developmental Disorders (F80-F89) | 1.72% (13353) | 1.14% (8811) | 1.08% (5380) | 0.85% (4221) |
| Pervasive Developmental Disorders (F84) | 0.17% (1355) | 0.11% (821) | 0.07% (344) | 0.05% (239) |
| Childhood Onset Disorders (F90-F98) | 2.75% (21272) | 2.87% (22225) | 1.84% (9146) | 2.19% (10857) |
| Hyperkinetic Disorder (F90) | 0.96% (7426) | 0.5% (3883) | 0.59% (2924) | 0.29% (1428) |

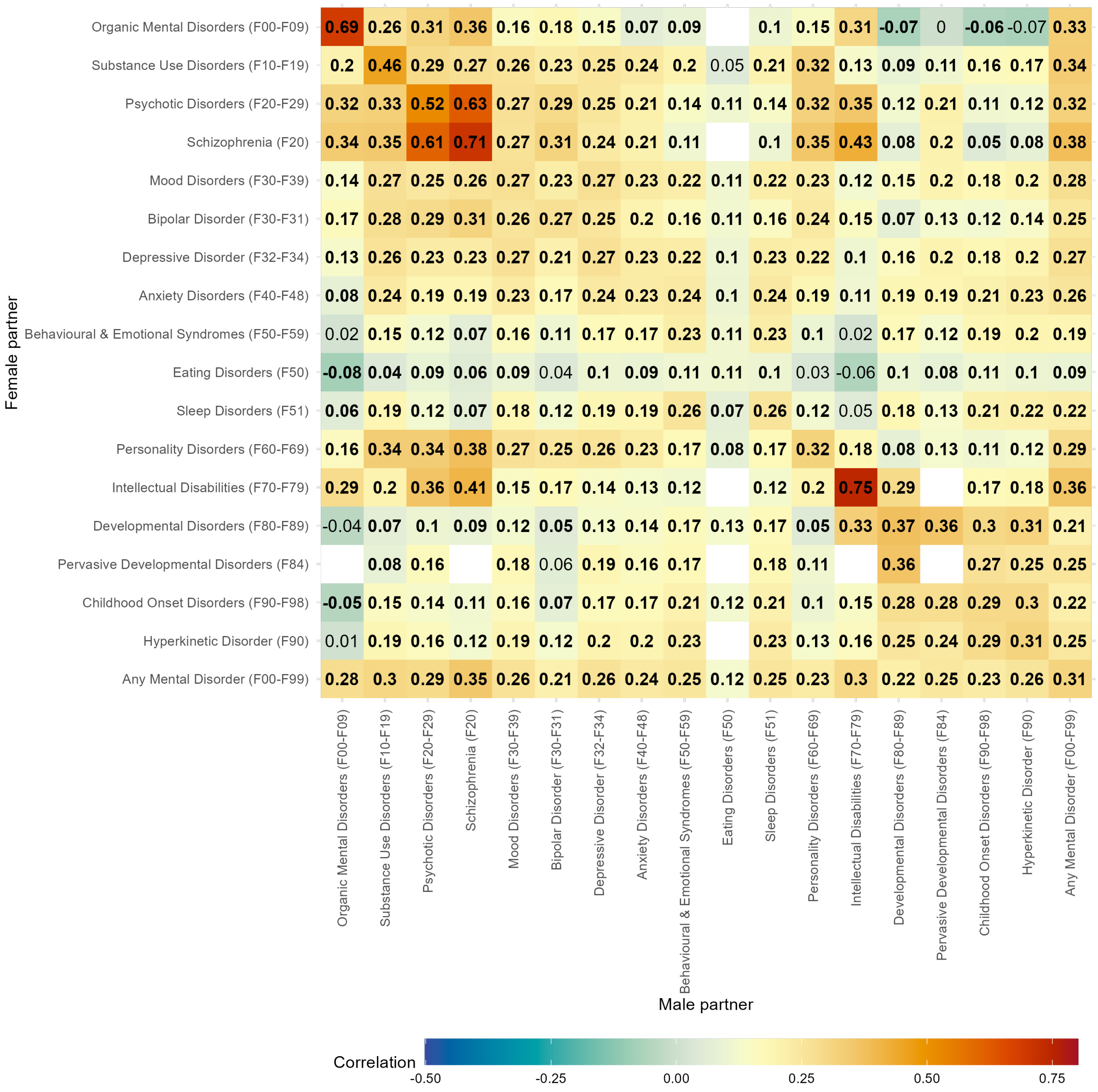


**Supplementary Figure 1.** Tetrachoric correlations within and between mental diagnoses of partners without children.

Estimates in bold indicate a statistically significant correlation (Bonferroni corrected p < 0.05). Empty values reflect the contingency table had an observed cell frequency of 0 or an expected cell frequency of less than 5.

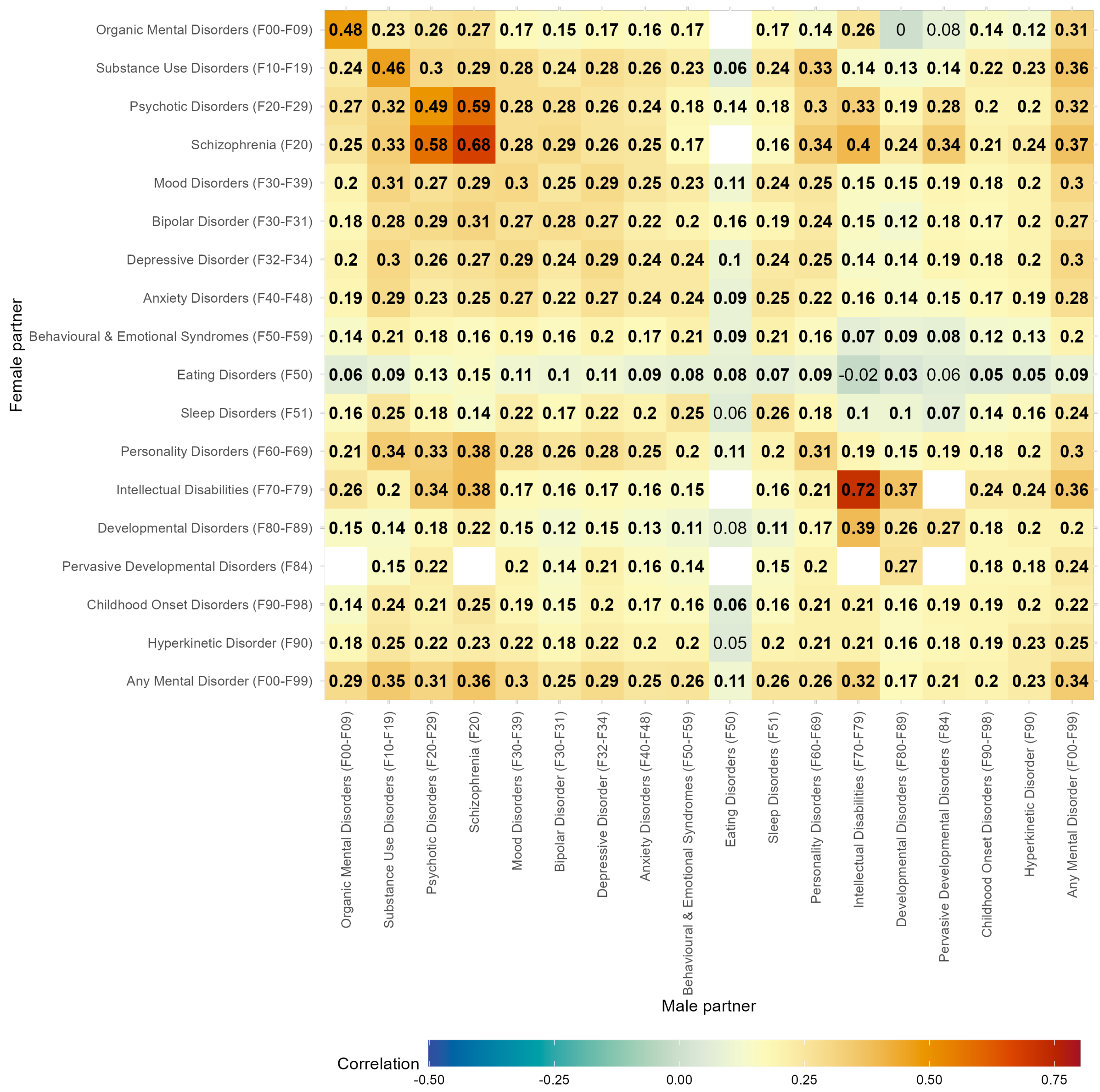


**Supplementary Figure 2.** Tetrachoric correlations within and between mental diagnoses of partners adjusted for a birth decade.

Estimates in bold indicate a statistically significant correlation (Bonferroni corrected p < 0.05). Empty values reflect the contingency table had an observed cell frequency of 0 or an expected cell frequency of less than 5.

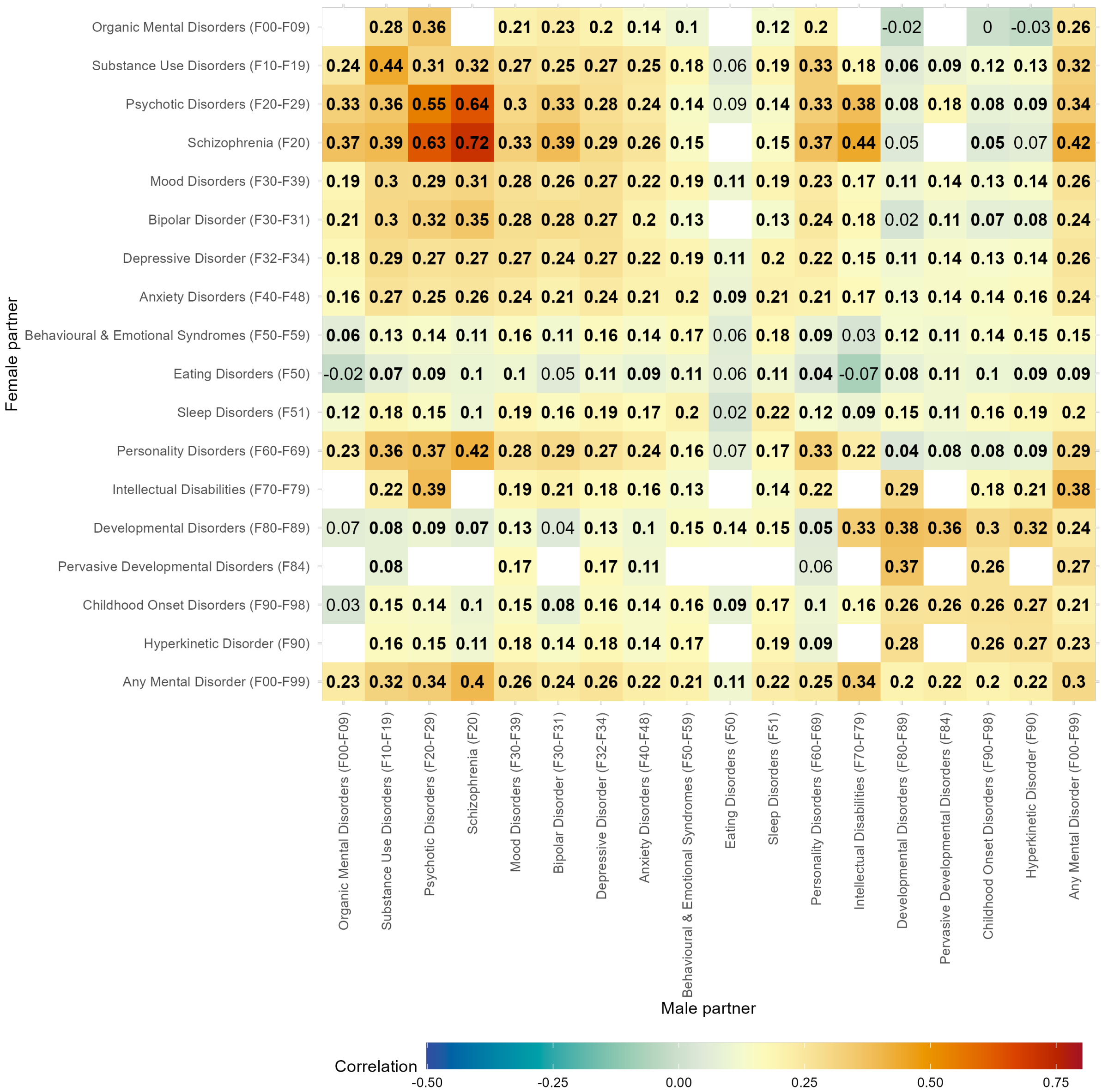


**Supplementary Figure 3.** Tetrachoric correlations within and between mental diagnoses of partners including only diagnoses 5 years before cohabitation.

Estimates in bold indicate a statistically significant correlation (Bonferroni corrected p < 0.05). Empty values reflect the contingency table had an observed cell frequency of 0 or an expected cell frequency of less than 5.

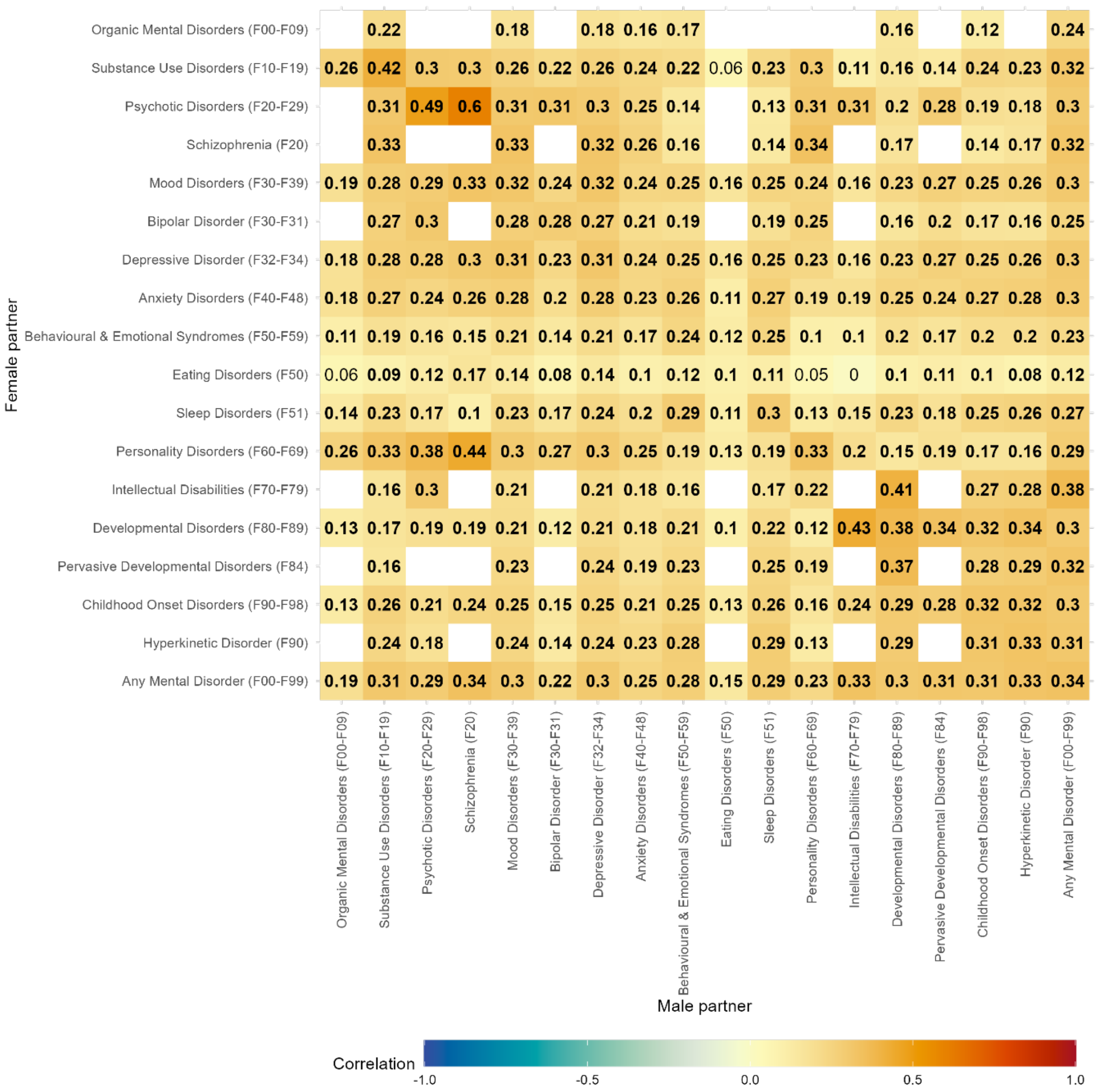


**Supplementary Figure 4.** Tetrachoric correlations within and between mental diagnoses of partners in the first recorded relationship.

Estimates in bold indicate a statistically significant correlation (Bonferroni corrected p < 0.05). Empty values reflect the contingency table had an observed cell frequency of 0 or an expected cell frequency of less than 5.

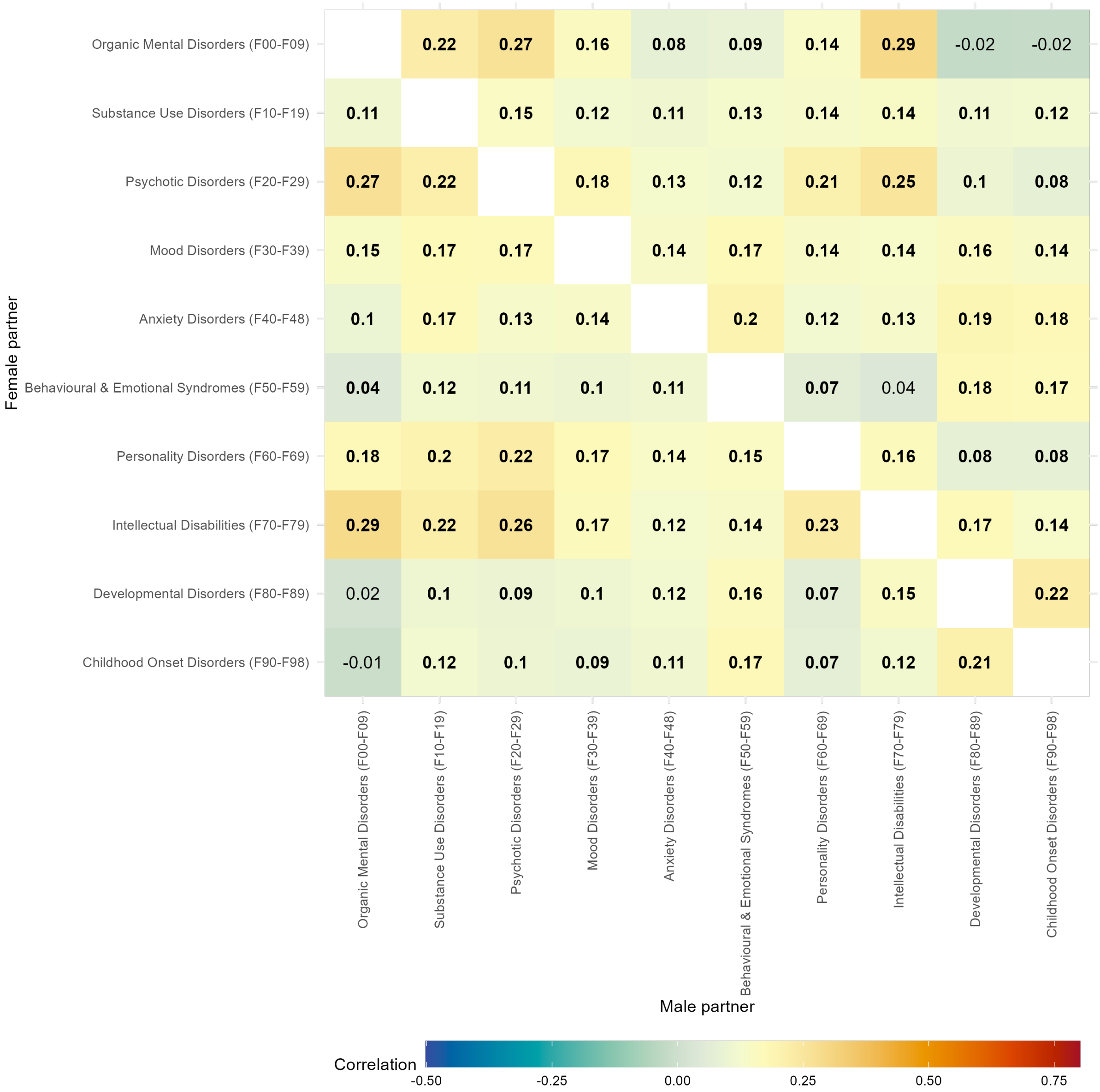


**Supplementary Figure 5.** Tetrachoric correlations within and between mental diagnoses of partners when comorbidities were excluded.

*Note*. Empty cells indicate that within-disorder analyses could not be conducted because all women diagnosed with the male partners' disorder of interest were excluded and all men diagnosed with the female partners' disorder of interest were also excluded. Estimates in bold indicate a statistically significant correlation (Bonferroni corrected $p < 0.05$).
